# Effects of 40% of Maximum Oxygen Uptake Intensity Cycling Combined with Blood Flow Restriction Training on Body Composition and Serum Biomarkers of Chinese College Students with Obesity

**DOI:** 10.3390/ijerph19010168

**Published:** 2021-12-24

**Authors:** Yong Chen, Chunlin Ma, Junmin Wang, Ying Gu, Yan Gao

**Affiliations:** 1 Department of Physical Education, Huaiyin Normal University, Huaian 223300, China; chenyong@hytc.edu.cn (Y.C.); 8200411008@hytc.edu.cn (C.M.); 8201911016@hytc.edu.cn (J.W.); 2 College of Sports Science, Shenyang Normal University, Shenyang 110034, China; 3 School of Foreign Languages, Shenyang Normal University, Shenyang 110034, China; 2016150197@jou.edu.cn

**Keywords:** 40% of maximum oxygen uptake, cycling, blood flow restriction training, male college students, obesity, body composition, serum biomarkers

## Abstract

Blood flow restriction training (BFRT) is a new method for promoting muscle growth and improving muscle function, even with relatively low-intensity exercise. BFRT on patients with obesity has not been extensively studied. This study aimed to analyze the effects of cycling at 40% of maximum oxygen uptake (VO_2_max) combined with BFRT on body composition and serum biomarkers among college students with obesity. This pilot study included thirty-seven male college students with obesity aged 18–22 years (experimental group (EG): *n* = 18; control group (CG): *n* = 19). The EG conducted 40% VO_2_max cycling combined with BFRT activities and the CG conducted 40% VO_2_max cycling without BFRT two times per week for 12 weeks. Our results showed that in EG, there were significant differences in weight, thigh skinfold thickness (TS), waist circumference, abdominal skinfold thickness, fat mass, body fat percentage, body mass index and glucose (GLU), total cholesterol (TC), triglyceride, low-density lipoprotein cholesterol (LDL-C), and high-density lipoprotein cholesterol (HDL-C) levels before and after the experiment (*p* < 0.05, *p* < 0.01, and *p* < 0.001). After the experiment, TS, GLU, TC, HDL-C, and LDL-C in EG were significantly different than those of the CG (*p* < 0.05, *p* < 0.01, and *p* < 0.001). Together, our results demonstrate that cycling at 40% VO_2_max combined with BFRT may improve body composition and blood lipid profile of male college students with obesity. Our findings have important implications for those who cannot perform moderate- and high-intensity exercises.

## 1. Introduction

Blood flow restriction training (BFRT) is a complementary exercise that is based on using a specific compression device combined with general exercise. It induces muscle ischemia in the distal limbs through pressure and is a new method for promoting muscle growth and improving muscle function, even with relatively low-intensity exercise [1]. This method, originally developed by Dr. Yoshiaki Sato in Japan, trains the muscles using specialized, pressurized equipment called KAATSU. Both KAATSU training and BFRT lead to ischemic characteristics. Because of its simple, time-saving, and effective features, BFRT is a popular technique. Currently, it is mainly used for sports training, mass fitness, and rehabilitation. Some gyms, fitness centers, hospitals, and rehabilitation centers in Japan and the United States are currently offering BFRT [1,2].

The research conducted by Abe 2006 (Eighteen males, Kaatsu-walk training, two times a day, six days/week for three weeks) [3], Sakuraba (Twenty-one athletes, BFRT-isokinetic resistance training, two times/week for three weeks) [4], Abe 2010 (Nineteen young males, Low-Intensity Cycle Training with BFRT, three times/week for eight weeks) [5], Sakamaki (Eight eumenorrheic females and five males, BFRT-30% one Repetition Maximum[RM] resistance training, one time/day for six days) [6], Yasuda (Ten young males, BFRT-30% 1RM resistance training, three days/week for six weeks) [7], and Wilson (Twenty male participants aged 21 ± 3 years, BFRT-30% 1RM resistance training. The participants completed a total of 5 testing sessions separated by a minimum of 72 hours’ rest over a 2- to 3-week period.) [8] show that BFRT has been reported to increase muscle volume. Research conducted by Yasuda (Ten young males, BFRT-30% 1RM resistance training, three days/week for six weeks) [7], Kacin (Ten healthy males, BFRT-15% maximal voluntary muscle contraction [MVC], four sessions/week for four weeks) [9], Madarame (Fifteen males, BFRT-50% 1RM resistance training, three sessions/time, two times/week for eight weeks) [10], Manimmanakorn (Thirty Well-trained netball players, BFRT-20% 1RM resistance training, one time/day for five days) [11], and Yasuda (Forty young males divided into four groups, 75% 1RM resistance training, BFRT-30% 1RM resistance training, 75% 1RM and BFRT-30% 1RM, non-training, three times/week for six weeks) [12] show that BFRT has been reported to increase the muscle cross-sectional area. Research conducted by Kubota (Fifteen healthy males, the left ankle of each male was immobilized, BFRT-walk using crutches with non–weight-bearing, two sessions/day for 14 days) [13], and Yamanaka (Thirty-two football players, BFRT-20% 1RM resistance training, three times/week for four weeks) [14], show that BFRT has been reported to increase muscle girth, as assessed using magnetic resonance imaging or ultrasound [3,4,5,6,7,8,15].

Previous studies have shown that BFRT can activate the mammalian target of rapamycin complex 1 (mTORC1) signaling pathway in the muscles of male individuals and stimulate protein synthesis [16]. Using muscle biopsy, the isotopic tracer method, and immunoblotting, Fry et al. [17] found that BFRT can significantly activate mTORC1 signal transduction and stimulate the protein synthesis of older individuals (70 ± 2 years). It was demonstrated, using muscle biopsy [16,17,18], immunohistochemistry, and protein expression techniques [16,17], that BFRT may promote muscle protein synthesis and/or inhibit protein breakdown.

The main mechanism underlying BFRT may be an increase in metabolic stress, including hormone secretion, regulation, and the inhibition of protein synthesis, muscle fiber mobilization, and cell swelling. In those unable to perform high-intensity strength training, BFRT is a good alternative for strength development [19]. More lean muscle mass likely increases the body’s basal metabolic rate, thus achieving the effect of consuming more fat [20]. Human experiments have shown that cell swelling not only inhibits proteolytic metabolism but also promotes lipolysis through protein sparing [21].

Low-intensity training with BFRT can stimulate muscle growth and improve muscle mass. Moreover, BFRT is not limited to a single training modality but can be performed combined with aerobic cycling or walking [1,22]. Due to the low physical load on muscles during BFRT, the recovery time after training is short [23] and muscle injury is minor [22]. Studies have shown that sports injuries are related to decreases in muscle strength caused by age, especially in women and patients with chronic diseases [24]. Therefore, BFRT can be particularly effective at improving muscle performance while reducing injury risk.

Although BFRT has attracted attention worldwide, it was mainly studied and used among people without training experience, undergoing recovery, or high-level athletes. The effects of BFRT on patients with obesity have not been extensively studied. Thus, due to its characteristics of low intensity, high efficiency, simplicity, and low risk of injury, we hypothesized that BFRT is suitable for people with obesity who cannot perform high-intensity exercise. Low-intensity BFRT should positively affect fat loss, body composition, and exercise ability in people with obesity. Therefore, we investigated the influence of low-intensity BFRT on muscle and fat in people with obesity by studying patients with obesity undergoing low-intensity BFRT as an intervention.

## 2. Materials and Methods

### 2.1. Study Participants

Among male college students with obesity in Jiangsu Province, China, forty male college students were recruited with a body fat percentage (%BF) > 25% or a body mass index (BMI) > 28 kg/m^2^ [25] and volunteered to participate in the study. Participants were randomly divided into the BFRT (experimental group [EG]) and general training (control group [CG]) groups using IBM SPSS Statistics for Windows software. With three participants withdrawing from the study midway, a total of 37 participants completed the experiment (EG, *n* = 18; CG, *n* = 19). Participants’ physical characteristics are shown in Table 1. None of the subjects had participated in regular strength, resistance, and/or aerobic training (less than once a week) for a minimum of 1 year prior to the start of the study. Participants with any of the following conditions were excluded from the study: (I) diabetes, angina pectoris, or hypertension; or (II) sports contraindications such as bone and joint disease or heart disease. After fully explaining the purpose and process of this study to the participants, informed consent was obtained prior to starting this study, with an option to opt-out at any time if they felt physically or psychologically tired or uncomfortable, following approval by the Ethics Committee of the Suzhou University (approval number: HR 106-2020).

Before the intervention, an independent-sample t-test was used to assess the physical characteristics of the EG and CG participants for homogeneity (Table 1). As there were no significant differences in age, height, weight, % BF, and BMI between the groups (all *p* > 0.05), homogeneity was assured.

### 2.2. Study Design

**Figure 1:**
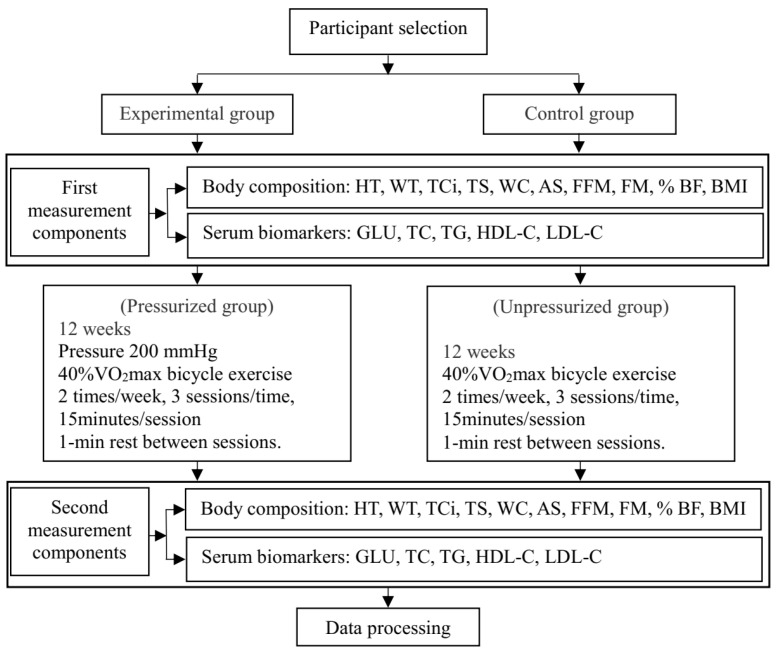
Study Procedures (HT, height; WT, weight; TCi, thigh circumference; TS, thigh skin fold thickness; WC, waist circumference; AS, abdominal skinfold thickness; FFM, fat-free mass, FM, fat mass; % BF, body fat percentage; BMI, body mass index; GLU, glucose; TC, Total cholesterol; TG, triglyceride; HDL-C, high-density lipoprotein cholesterol; LDL-C, Low-density lipoprotein cholesterol).

A total of 24 times (2 times/week, 12 weeks), participants performed low-intensity cycling combined with BFRT exercises (EG) and low-intensity cycling without BFRT exercises (CG). The body composition and serum markers of the EG group and the CG group were measured before and after the intervention. The design of this study is shown in Figure 1.

### 2.3. Intervention Protocol

The participants performed once a day, 2 days/week, for 12 weeks. Following measurements of body composition and serum biomarker, the participants performed exercises on an electrically braked bicycle ergometer (Aerobike 800, Combi Corporation, Tokyo, Japan) at a predetermined 40% of VO_2_max combined with BFRT for 45 min (3 sessions, 15 min/session, 1-min rest between session) in the EG group and at a predetermined 40% of VO_2_max for 45 min (3 sessions, 15 min/session, 1-min rest between session) in the CG group. Throughout the intervention period, each group’s exercise intensity and duration remained unchanged. Participants in the EG group wore air pressure belts (Kaatsu-Master, Sato Sports Plaza, Tokyo, Japan) on the base of both thighs during cycle exercise training. The choice of 160–200 pressure is based on a pilot study of young men [3]. During each training, the blood flow to the leg muscles is restricted for a total of about 50 min (3 min of preparation time, 3 sessions of 15 min of cycling time per session and 2 rests of 1 min), and the belt pressure was released immediately after each session of exercises. One week before the intervention, the participants took a familiarization course (without BFRT). The machine settings (seats, leg pad) of each participant were recorded and standardized.

### 2.4. Data Collections

Body composition: Thigh circumference (TCi), thigh skinfold thickness (TS), waist circumference (WC), and abdominal skinfold thickness (AS) was measured by the same researcher using a JK6113 skin thickness meter and measuring tape (Beijing Jingkaida Instrument Co., Ltd., Beijing, China). Waist circumference was measured at the midpoint between the lower margin of the last palpable rib and the iliac crest. Thigh circumference was measured while standing, foot on a bench, with the knee and hip flexed 90°. The measurement was taken at the midpoint between the most proximal thigh, the intersection of the inguinal crease and the anterior midline of the thigh, and the proximal border of the patella. Thigh skinfold thickness was measured in the middle of the hip joint and knee joint, and the front of the thigh was longitudinally pinched to measure the skinfold along the long axis of the trunk. Abdominal skinfold thickness was measured at the junction of the horizontal line of the umbilicus and the midline of the right clavicle, and the skinfold was measured longitudinally along the long axis of the trunk. All girth and thickness were measured twice and averaged. If the 2 measurements were not within 5 mm, a third measurement was taken [26]. Fat-free mass (FFM), fat mass (FM), % BF, and BMI were measured using the X-Scan Plus II body composition analyzer (ACCUNIQ Company of Korea). The measurement was to be carried out on an empty stomach or 2 to 3 h after eating and standing for 5 min before the measurement. Participants were told not to bring heavy objects or metal ornaments. During the measurement, the subject stood barefoot on the detector. They were told to wear as few, light clothes as possible and to fully touch the electrodes with both hands and feet.

Serum biomarkers: Including glucose (GLU), total cholesterol (TC), triglyceride (TG), high-density lipoprotein cholesterol (HDL-C), and low-density lipoprotein cholesterol (LDL-C) levels were measured. Participants fasted after 8 o’clock in the evening of the previous day. After fasting (≥12 h), blood was obtained from the anterior elbow vein by a trained phlebotomist from 6 am to 8 am the next morning. After 30 min, the tubes were centrifuged at 2100× *g* for 10 min at 4 °C. The serum was stored at −80 °C until the study was completed for batch analysis. Serum was analyzed using the AU5800-10 automatic clinical chemical analyzer (Beckman Coulter, Inc., Brea, CA, USA).

### 2.5. Statistical Analysis

All data in this study were analyzed using IBM SPSS Statistics for Windows software (2017, v 23.0, Armonk, NY, USA: IBM Corp.). After the intervention, the measured data of the EG and CG were analyzed using mean values and standard deviation. Statistical analyses were performed using a two-way analysis of variance (ANOVA) with repeated measures (Group [EG vs. CG] × Time [pre vs. post]) to evaluate training effects for all dependent variables. A paired-sample t-test was conducted to analyze data before and after the intervention in both groups. Additionally, a t-test was used to analyze changes in the EG and CG before and after the intervention. All *p* < 0.05 were considered statistically significant.

## 3. Results

Two-way repeated measurement analysis of variance method was used to judge the influence of different interventions on various physical indicators of the subjects over time. Through the analysis of studentized residuals and the Shapiro-Wilk test, each group of data obeys a normal distribution (*p* > 0.05); judging by whether the studentized residual exceeds ±3 times the standard deviation, each group of data had no outliers. After Mauchly’s test of sphericity, for the interaction term group × time, the variance and covariance matrices of the dependent variables were equal (*p* > 0.05).

### 3.1. Body Composition Results

After the intervention, in the EG group, WT, WC, FM, and% BF decreased significantly compared with the CG group (*p* < 0.05) (Table 2). In the EG, there was a significant difference in WT, TS, WC, AS, FM, % BF, and BMI before and 12 weeks after the intervention (*p* < 0.05 and *p* < 0.01, respectively). In the CG, there were no significant differences in all indicators before and after the intervention (Table 3).

### 3.2. Serum Biomarker Results

After the intervention, in the EG group, GLU, TC, and LDL-C decreased significantly compared with the CG group (*p* < 0.05). HDL-C increased significantly compared with the CG group (*p* < 0.05) (Table 4). In the EG, there was a significant difference in GLU, TC, TG, HDL-C, and LDL-C levels before and 12 weeks after the intervention (*p* < 0.05 and *p* < 0.001, respectively). In the CG, there was also a significant difference in GLU, TC, and LDL-C levels before and after the intervention (*p* < 0.05) (Table 5).

## 4. Discussion

According to the results of this study, TS, WC, AS, FM, % BF, and BMI changed significantly in the EG after the intervention compared to those before the intervention, and no change was seen among the CG. The results of this study are consistent with the results of similar studies. For example, a study by Fei [27] on healthy men aged 30–45 years showed that BFRT significantly reduced WT, BMI, FM, and body age. In the CG, there were no significant changes in BMI and FM before and after the intervention. The results of the present study show that cycling exercise at 40% VO_2_max combined with BFRT could significantly reduce FM, % BF, BMI, and subcutaneous fat thickness in people with obesity, as well as optimize their body composition.

Zhouming [28] conducted an aerobic exercise intervention at 55–85% VO_2_max on college students for 12 weeks. The % BF and BMI of male college students with low VO_2_max significantly decreased. This confirmed that cycling exercise at 40% VO_2_max combined with BFRT is a better choice for people with obesity (due to lower intensity) compared with aerobic exercise at approximately 55–85% VO_2_max without blood flow restriction (BFR).

Conventionally, it is understood that aerobic exercise does not cause muscle hypertrophy [29] and even weakens the muscle hypertrophy effect of strength training when performed in parallel with strength training [30]. However, in this intervention, TS in the EG significantly decreased, while TC did not significantly change, suggesting that thigh muscle swelling occurred with a decrease in subcutaneous fat in the thighs. This finding is consistent with that of similar studies, such as Abe et al. [5,12], suggesting that BFRT combined with low-intensity aerobic exercise (walking and cycling) can cause muscle hypertrophy. Abe et al. [5] conducted a bike training experiment on 19 men for eight weeks and showed that the EG with BFRT (15 min/session) led to a significantly increased cross-sectional area in the thigh muscles and maximum voluntary contraction (MVC) following the experiment compared with the values before training, while the CG (45 min/session) had no significant changes. It is suggested that low-intensity BFRT can also cause muscle hypertrophy in people with obesity. Various studies have shown that BFRT increases the muscle volume and strength of athletes [14,31], weightlifters [32], and non-athletes [33]. Therefore, this indicates that low-intensity BFRT can improve the muscle strength and exercise ability, as well as the muscle hypertrophy, of male college students with obesity.

Human experiments have shown that cell swelling caused by BFRT not only inhibits protein catabolism but also has a positive effect on protein synthesis through protein-sparing; it also promotes lipolysis [21].

Although there is extensive evidence that BFRT can promote muscle strength and hypertrophy, it is unclear whether cell swelling is caused by stress or the combination of the interaction of stress and low-intensity exercise. Moreover, the similarities and differences between the effects of low-intensity blood flow restriction resistance exercise and BFR aerobic exercise on the human body are also unclear. Furthermore, whether fat loss caused by low-intensity BFRT is mainly due to the protein-saving effect and promotion of fat lipolysis or excessive energy consumption due to BFR has not been fully elucidated.

However, in this study, TS, WC, AS, FM, % BF, and BMI tended to significantly decrease in the EG, suggesting that BFRT can promote lipolysis.

In this intervention, GLU, TC, TG, HDL-C, and LDL-C in the EG, showed significant differences before and after the intervention (*p* < 0.05 and *p* < 0.001, respectively). After 12 weeks, there were significant differences in GLU, TC, HDL-C, and LDL-C levels in the EG compared with the levels in the CG (*p* < 0.05, *p* < 0.01, and *p* < 0.001, respectively). The effects of BFRT on TC, TG, HDL-C, LDL-C, and blood GLU levels have not been systematically reported. According to a study by Wei et al. [34], low-intensity resistance training (at 20% 1RM intensity) combined with appropriate exercise can significantly reduce fasting blood GLU and insulin levels through blood flow restriction. Simultaneously, it was found that serum adiponectin levels significantly increased following exercise, and this is consistent with the results showing a significant decrease in GLU in EG found in this study.

According to the Resistance Exercise Training guidelines provided by the American Heart Association in 2011, Williams et al. [35] evaluated the effect of anti-resistance training on blood lipid levels. They concluded that aerobic exercise was superior to resistance exercise in terms of pure improvement of blood lipid levels. Thus, resistance exercise should be a beneficial supplement to aerobic exercise, characterized by the effective development of muscle strength and changes in body composition. In the present study, it was shown that cycling exercise at 40% VO_2_max intensity combined with BFRT had characteristics of aerobic exercise and a greater effect on blood lipids than cycling exercise at 40% VO_2_max unrestricted blood flow.

The blood flow restriction test of Fei [27] used a 20% 1RM blood flow restriction resistance test, which did not show the expected significant changes in HDL-C and LDL-C levels. In the present intervention, cycling exercise at 40% VO_2_max intensity combined with BFRT was used. The differences between the results of the aforementioned study and ours may be due to differences in exercise mode and intensity. In the BFRT study, there are few studies that discuss HDL-C, but all studies focusing on the effect of exercise on HDL-C seem to consistently indicate that there was an increase in HDL-C mor, no matter in humans or rats [36]. What is interesting is that both aerobic exercise [37] and strenuous exercise [38] are helpful in improving HDL-C. A study by Wang [36] found that HDL-C levels are more sensitive to aerobic exercise than LDL-C and TG. In this intervention, the significant changes in all serum biomarkers may be attributed to the fact that aerobic exercise with low-intensity blood flow restriction can achieve the same effect as long-term exercise and that increases in exercise volume will improve the energy metabolism of the body.

Studies have shown that cycling exercise at 40% VO_2_max intensity leads to a higher proportion of adipose tissue oxidation for energy than that at 65% VO_2_max intensity [39]. However, in the present intervention, when the EG cycled at 40% VO_2_max intensity combined with BFRT, there were significant differences in all instances before and after the intervention, both in intra- and inter-group comparisons. Results have shown that cycling exercise at 40% VO_2_max combined with BFRT could effectively promote the lipolysis and energy consumption for obese people to participate in exercises. Simultaneously, compared with cycling exercises at 40% VO_2_max combined without BFRT, BFRT can promote the oxidative energy supply of adipose tissue. Combined with BMI, WT, % BF, TC, TG, and other indexes, BFRT can improve body composition and lipid metabolism and promote overall fitness by reducing fat and controlling body weight in patients with obesity.

Regarding the mechanism underlying the improvement of energy metabolism by BFRT, it is necessary to increase the determination of LPL and related metabolic factors, such as mitochondria, to further understand this mechanism.

### 4.1. Limitations

The participants in this research were young people. It cannot be guaranteed that the results of this experiment are equally effective for the elderly with obesity. Although studies have shown that BFRT combined with resistance exercise can effectively reduce blood pressure in the elderly [40], BFRT combined with walking can effectively increase the cross-sectional muscle area of the elderly [41], and BFRT combined with endurance exercise can increase aerobic metabolism and basic consumption [42]. However, as the metabolism of the elderly slows down, various organs decline with age, and the ability to adapt to environmental changes also decreases. Therefore, the results of this study may not be applicable to the elderly with obesity, but further verification is needed. Prior studies have shown that BFRT combined with resistance exercises can effectively improve muscle mass and maximum oxygen uptake [43], while BFRT combined with aerobic exercises can also effectively improve muscle mass and maximum oxygen uptake [5,44]. Aerobic exercise [37] and strenuous exercise [38] can improve HDL-C, showing that BFRT combined with resistance exercise and aerobic exercise can improve muscle mass, increase maximum oxygen uptake, and improve blood lipids. However, BFRT combined with resistance exercise is better than BFRT combined with walking exercises on muscle strength and muscle hypertrophy [45]. BFRT combined with high-intensity resistance exercise may have a greater impact on blood pressure than BFRT combined with low-intensity resistance exercise [46]. Therefore, we suggest that BFRT combined with resistance exercise and BFRT combined with aerobic exercise have different effects on the body. For different purposes, different types of exercise should be used. Therefore, it cannot be considered that resistance exercise combined with BFRT under the same intensity can produce the same effect as this experiment.

### 4.2. Practical Applications

Low-intensity aerobic exercise combined with BFRT can optimize the body composition of obese patients. Therefore, for the purpose of reducing fat, controlling weight, and improving muscle mass, it is recommended to use aerobic exercise combined with BFRT as an auxiliary exercise in the fitness process. Because BFRT can promote muscle hypertrophy, it can also be used as an auxiliary method in the stage of improving strength. In addition, because BFRT requires less intensity, it is a good choice for people who have little strength and cannot perform moderate and high-intensity exercises. BFRT can have a positive effect on the serum biomarkers of obesity, and the requirement for exercise intensity is very low. Therefore, those who have high blood lipid levels and lack exercise time can use BFRT appropriately in their daily life and work.

## 5. Conclusions

Cycling exercise at 40% VO_2_max combined with BFRT can improve the body composition and lipid metabolism of patients with obesity, promote overall fitness, reduce body fat, and control weight. It is important for those who cannot perform moderate- and high-intensity exercise to improve their health and exercise ability.

## Figures and Tables

**Table 1:** Participants’ physical characteristics and homogeneity test for physical characteristics.

Variable	EG (*n* = 18)	CG (*n* = 19)	Levene’s Test		*t*-Test	
	(M ± SD)		F	*p*	*t*	*p*
Age (years)	20.3 ± 1.07	20.1 ± 1.08	0.155	0.696	0.636	0.529
Height (cm)	171.6 ± 3.95	170.2 ± 3.84	0.000	0.984	1.100	0.278
Weight (Kg)	88.7 ± 5.06	87.7 ± 4.60	0.678	0.416	0.645	0.523
% BF (%)	28.7 ± 1.18	28.9 ± 1.29	0.401	0.531	−0.319	0.752
BMI (Kg/m^2^)	30.1 ± 0.95	30.3 ± 1.08	0.538	0.468	−0.474	0.639

EG: experimental group; CG: control group; M: mean; SD: standard deviation; % BF: body fat percentage; BMI: body mass index.

**Table 2:** The influence of different interventions on body composition (two-way ANOVA).

Item	Between-Subject (Group)		Within-Subject (Time)		Interaction (Group × Time)	
	F (1, 35)	Partial η^2^	F (1, 35)	Partial η^2^	F (1, 35)	Partial η^2^
WT (kg)	0.000	0.000	43.627 *	0.555	9.713 *	0.271
TCi (cm)	0.089	0.003	7.247 *	0.172	0.133	0.004
TS (mm)	3.818	0.098	15.419 *	0.555	0.275	0.073
WC (cm)	0.349	0.010	7.118 *	0.169	4.327 *	0.110
AS (mm)	1.041	0.029	5.096 *	0.127	2.789	0.074
FFM (kg)	0.185	0.005	6.270 *	0.152	0.691	0.019
FM (kg)	0.346	0.010	22.179 *	0.388	8.434 *	0.194
% BF (%)	1.904	0.030	11.750 *	0.251	12.613 *	0.265
BMI (kg/m^2^)	2.098	0.057	9.844 *	0.220	2.059	0.056

WT, weight; TCi, thigh circumference; TS, thigh skin fold thickness; WC, waist circumference; AS, abdominal skinfold thickness; FFM, fat-free mass, FM, fat mass; % BF, body fat percentage; BMI, body mass index; * *p* < 0.05.

**Table 3:** Changes of body composition of experimental and control groups before and after 12 weeks (*t*-test).

Body Composition Components	Group	Pre	Post	Pre-Post Difference	*t*
		(M ± SD)			
WT (kg)	EG (*n* = 18)	88.7 ± 5.06	85.5 ± 4.85	3.22 ± 5.46	2.50 *
	CG (*n* = 19)	87.7 ± 4.60	86.5 ± 4.97	1.15 ± 2.68	1.88
TCi (cm)	EG (*n* = 18)	60.6 ± 3.66	58.7 ± 3.74	1.94 ± 4.40	1.86
	CG (*n* = 19)	60.1 ± 2.72	58.6 ± 3.64	1.47 ± 3.25	2.00
TS (mm)	EG (*n* = 18)	27.8 ± 2.09	26.3 ± 2.25	1.56 ± 1.85	3.56 **
	CG (*n* = 19)	28.5 ± 1.83	27.9 ± 1.89	0.63 ± 1.53	1.80
WC (cm)	EG (*n* = 18)	97.6 ± 3.28	96.5 ± 2.90	1.11 ± 1.85	2.54 *
	CG (*n* = 19)	96.4 ± 3.70	96.3 ± 3.94	0.14 ± 0.82	0.73
AS (mm)	EG (*n* = 18)	54.9 ± 3.84	54.4 ± 3.60	0.53 ± 0.96	2.33 *
	CG (*n* = 19)	55.9 ± 3.99	55.9 ± 3.57	0.08 ± 0.65	0.53
FFM (kg)	EG (*n* = 18)	63.2 ± 3.14	61.4 ± 3.88	1.74 ± 4.09	1.81
	CG (*n* = 19)	62.3 ± 2.85	61.5 ± 3.24	0.87 ± 1.94	1.96
FM (kg)	EG (*n* = 18)	25.5 ± 2.24	24.0 ± 1.63	1.47 ± 1.46	4.27 **
	CG (*n* = 19)	25.3 ± 2.14	25.0 ± 2.11	0.35 ± 0.82	1.86
% BF (%)	EG (*n* = 18)	28.7 ± 1.18	28.1 ± 1.38	0.59 ± 0.59	4.25 **
	CG (*n* = 19)	28.9 ± 1.29	28.9 ± 1.28	0.01 ± 0.43	−0.11
BMI (kg/m^2^)	EG (*n* = 18)	30.1 ± 0.951	29.0 ± 1.45	1.06 ± 1.79	2.51 *
	CG (*n* = 19)	30.3 ± 1.08	29.9 ± 1.44	0.40 ± 0.92	1.88

EG: Experimental Group; CG: Control Group; WT, weight; TCi, thigh circumference; TS, thigh skin fold thickness; WC, waist circumference; AS, abdominal skinfold thickness; FFM, fat-free mass, FM, fat mass; % BF, body fat percentage; BMI, body mass index; * *p* < 0.05; ** *p* < 0.01.

**Table 4:** The influence of different interventions on serum biomarkers (two-way ANOVA).

Item	Between-Subject (Group)		Within-Subject (Time)		Interaction (Group × Time)	
	F (1, 35)	Partial η^2^	F (1, 35)	Partial η^2^	F (1, 35)	Partial η^2^
GLU (mmol/L)	5.282 *	0.131	100.003 *	0.741	41.002 *	0.539
TC (mmol/L)	10.296 *	0.227	77.642 *	0.689	48.621 *	0.581
TG (mmol/L)	0.166	0.005	9.530 *	0.214	2.498	0.067
HDL-C (mmol/L)	0.925	0.026	16.049 *	0.314	21.881 *	0.385
LDL-C (mmol/L)	3.584	0.093	239.654 *	0.873	148.409 *	0.809

GLU, glucose; TC, Total cholesterol; TG, triglyceride; HDL-C, high-density lipoprotein cholesterol; LDL-C, Low-density lipoprotein cholesterol; * *p* <0.05.

**Table 5:** Changes of serum biomarkers of experimental and control groups before and after 12 weeks (*t*-test).

Serum Biomarkers	Group	Pre	Post	Pre-Post Difference	*t*
		(M ± SD)			
GLU (mmol/L)	EG (*n* = 18)	5.36 ± 0.30	4.92 ± 0.29	0.43 ± 0.17	11.00 ***
	CG (*n* = 19)	5.39 ± 0.25	5.29 ± 0.26	0.10 ± 0.16	2.68 *
TC (mmol/L)	EG (*n* = 18)	4.99 ± 0.20	4.59 ± 0.27	0.41 ± 0.21	8.38 ***
	CG (*n* = 19)	5.01 ± 0.17	4.96 ± 0.16	0.05 ± 0.09	2.36 *
TG (mmol/L)	EG (*n* = 18)	1.27 ± 0.19	1.20 ± 0.16	0.08 ± 0.11	2.75 *
	CG (*n* = 19)	1.28 ± 0.25	1.25 ± 0.23	0.02 ± 0.079	1.34
HDL-C (mmol/L)	EG (*n* = 18)	1.50 ± 0.22	1.71 ± 0.17	0.14 ± 0.13	−4.36 ***
	CG (*n* = 19)	1.51 ± 0.20	1.50 ± 0.20	0.01 ± 0.03	1.40
LDL-C (mmol/L)	EG (*n* = 18)	2.55 ± 0.56	1.98 ± 0.54	0.57 ± 0.11	2.08 ***
	CG (*n* = 19)	2.61 ± 0.43	2.55 ± 0.49	0.07 ± 0.13	2.22 *

EG: Experimental Group; CG: Control Group; GLU, glucose; TC, Total cholesterol; TG, triglyceride; HDL-C, high-density lipoprotein cholesterol; LDL-C, Low-density lipoprotein cholesterol; * *p* < 0.05; *** *p* < 0.001.

## Data Availability

Data provided in this study are available upon request by the corresponding author and the first author. The data were not made public because it involves the personal privacy issues of the participants.

## References

[1] Sato Y. (2005). The history and future of KAATSU training. Int. J. KAATSU Train. Res..

[2] Loenneke J.P., Pujol T.J. (2009). The use of occlusion training to produce muscle hypertrophy. Strength Cond. J..

[3] Abe T., Kearns C.F., Sato Y. (2006). Muscle size and strength are increased following walk training with restricted venous blood flow from the leg muscle, Kaatsu-walk training. J. Appl. Physiol..

[4] Sakuraba K., Ishikawa T. (2009). Effect of isokinetic resistance training under a condition of restricted blood flow with pressure. J. Orthop. Sci..

[5] Abe T., Fujita S., Nakajima T., Sakamaki M., Ozaki H., Ogasawara R., Sugaya M., Kudo M., Kurano M., Yasuda T. (2010). Effects of low-intensity cycle training with restricted leg blood flow on thigh muscle volume and VO_2_max in young men. J. Sports Sci. Med..

[6] Sakamaki M., Yasuda T., Abe T. (2012). Comparison of low-intensity blood flow-restricted training-induced muscular hypertrophy in eumenorrheic women in the follicular phase and luteal phase and age-matched men. Clin. Physiol. Funct. Imaging.

[7] Yasuda T., Loenneke J.P., Thiebaud R.S., Abe T. (2012). Effects of blood flow restricted low-intensity concentric or eccentric training on muscle size and strength. PLoS. ONE.

[8] Wilson J.M., Lowery R.P., Joy J.M., Loenneke J.P., Naimo M.A. (2013). Practical blood flow restriction training increases acute determinants of hypertrophy without increasing indices of muscle damage. J. Strength Cond. Res..

[9] Kacin A., Strazar K. (2011). Frequent low-load ischemic resistance exercise to failure enhances muscle oxygen delivery and endurance capacity. Scand. J. Med. Sci. Sports.

[10] Madarame H., Neya M., Ochi E., Nakazato K., Sato Y., Ishii N. (2008). Cross-transfer effects of resistance training with blood flow restriction. Med. Sci. Sports Exerc..

[11] Manimmanakorn A., Hamlin M.J., Ross J.J., Taylor R., Manimmanakorn N. (2013). Effects of low-load resistance training combined with blood flow restriction or hypoxia on muscle function and performance in netball athletes. J. Sci. Med. Sport.

[12] Yasuda T., Ogasawara R., Sakamaki M., Ozaki H., Sato Y., Abe T. (2011). Combined effects of low-intensity blood flow restriction training and high-intensity resistance training on muscle strength and size. Eur. J. Appl. Physiol..

[13] Kubota A., Sakuraba K., Sawaki K., Sumide T., Tamura Y. (2008). Prevention of disuse muscular weakness by restriction of blood flow. Med. Sci. Sports Exerc..

[14] Yamanaka T., Farley R.S., Caputo J.L. (2012). Occlusion training increases muscular strength in division IA football players. J. Strength Cond. Res..

[15] Loenneke J.P., Young K.C., Wilson J.M., Andersen J.C. (2013). Rehabilitation of an osteochondral fracture using blood flow restricted exercise: A case review. J. Bodyw. Mov. Ther..

[16] Fujita S., Abe T., Drummond M.J., Cadenas J.G., Dreyer H.C., Sato Y., Volpi E., Rasmussen B.B. (2007). Blood flow restriction during low-intensity resistance exercise increases S6K1 phosphorylation and muscle protein synthesis. J. Appl. Physiol..

[17] Fry C.S., Glynn E.L., Drummond M.J., Timmerman K.L., Fujita S., Abe T., Dhanani S., Volpi E., Rasmussen B.B. (2010). Blood flow restriction exercise stimulates mTORC1 signaling and muscle protein synthesis in older men. J. Appl. Physiol..

[18] Manini T.M., Vincent K.R., Leeuwenburgh C.L., Lees H.A., Kavazis A.N., Borst S.E., Clark B.C. (2011). Myogenic and proteolytic mRNA expression following blood flow restricted exercise. Acta Physiol..

[19] Jia W., Bo L., Wei Y., Xinxin W., Lianshi F., Yongming L. (2019). Application effect and mechanism of blood flow restriction training. China Sport Sci..

[20] Romijn J.A., Klein S., Coyle E.F., Sidossis L.S., Wolfe R.R. (1993). Strenuous endurance training increases lipolysis and triglyceride-fatty acid cycling at rest. J. Appl. Physiol..

[21] Keller U., Szinnai G., Bilz S., Berneis K. (2003). Effects of changes in hydration on protein, glucose and lipid metabolism in man: Impact on health. Eur. J. Clin. Nutr..

[22] Loenneke J.P., Abe T., Wilson J.M., Thiebaud R.S., Fahs C.A., Rossow L.M., Bemben M.G. (2012). Blood flow restriction: An evidence based progressive model (Review). Acta Physiol. Hung..

[23] Wernbom M., Järrebring R., Andreasson M.A., Augustsson J. (2009). Acute effects of blood flow restriction on muscle activity and endurance during fatiguing dynamic knee extensions at low load. J. Strength Cond. Res..

[24] Kerr Z.Y., Collins C.L., Comstock R.D. (2010). Epidemiology of weight training-related injuries presenting to United States emergency departments, 1990 to 2007. Am. J. Sports Med..

[25] Bei-Fan Z. (2002). Cooperative Meta-Analysis Group of Working Group on Obesity in China Predictive values of body mass index and waist circumference for risk factors of certain related diseases in Chinese adults: Study on optimal cut-off points of body mass index and waist circumference in Chinese adults. Asia Pac. J. Clin. Nutr..

[26] Walter R., Thompson American College of Sports Medicine (2009). ACSM’s Guidelines for Exercise Testing and Prescription.

[27] Fei W. (2015). Meta-Analysis and Empirical Study on the Effect of Compression Resistance Training on Cardiovascular System. Master’s Thesis.

[28] Zhouming C. (2017). Influence of Resistance and Aerobc Training on the Maximum Oxygen Uptake of College Students and the Mechanism of Exercise Adaptation. Ph.D. Thesis.

[29] Kraemer W.J., Patton J.F., Gordon S.E., Harman E.A., Deschenes M.R., Reynolds K., Newton R.U., Triplett N.T., Dziados J.E. (1995). Compatibility of high-intensity strength and endurance training on hormonal and skeletal muscle adaptations. J. Appl. Physiol..

[30] Bell G.J., Syrotuik D., Martin T.P., Burnham R., Quinney H.A. (2000). Effect of concurrent strength and endurance training on skeletal muscle properties and hormone concentrations in humans. Eur. J. Appl. Physiol..

[31] Abe T., Yasuda T., Midorikawa Y., Sato C.F., Kearns K., Inoue K., Koizumi N.I. (2005). Skeletal muscle size and circulating IGF-1 are increased after two weeks of twice daily ‘KAATSU’ resistance training. Int. J. KAATSU Train. Res..

[32] Godawa T.M., Credeur D.P., Welsch M.A. (2012). Influence of compressive gear on powerlifting performance: Role of blood flow restriction training. J. Strength Cond. Res..

[33] Madarame H., Ochi E., Tomioka Y., Nakazato K., Ishii N. (2011). Blood flow-restricted training does not improve jump performance in untrained young men. Acta Physiol. Hung..

[34] Wei C., Juan L., Qinghe C., Ningning S., Hongtao Y., Lei G. (2010). Effect of motor muscle flow restriction on body composition and insulin sensitivity in obese patients during resistance training. Chin. J. Sports Med..

[35] Williams M.A., Haskell W.L., Ades P.A., Amsterdam E.A., Bittner V., Franklin B.A., Gulanick M., Laing S.T., Stewart K.J., American Heart Association Council on Clinical Cardiology (2007). Resistance exercise in individuals with and without cardiovascular disease: 2007 update: A scientific statement from the American Heart Association Council on Clinical Cardiology and Council on Nutrition, Physical Activity, and Metabolism. Circulation.

[36] Wang Y., Xu D. (2017). Effects of aerobic exercise on lipids and lipoproteins. Lipids Health Dis..

[37] Nassef Y., Lee K.J., Nfor O.N., Tantoh D.M., Chou M.C., Liaw Y.P. (2019). The impact of aerobic exercise and badminton on HDL cholesterol levels in adult Taiwanese. Nutrients.

[38] Sarzynski M.A., Ruiz-Ramie J.J., Barber J.L., Slentz C.A., Apolzan J.W., McGarrah R.W., Harris M.N., Church T.S., Borja M.S., He Y. (2018). Effects of increasing exercise intensity and dose on multiple measures of HDL (high-density lipoprotein) function. Arterioscler. Thromb. Vasc. Biol..

[39] Yu L., Yuxiu H., Lifang Z., Jing Z., Xuguang L. (2006). Comparison of physiological and metabolic responses of obese male youth to two kinds of intensity aerobic exercise. China Sports Sci. Technol..

[40] Maciel A.W.S., Pinto L.M., Campos R.C.A., Ferreira A.C., Dias-Filho C.A.A., Dias C.J.M., Pires F.O., Urtado C.B., Rodrigues B., Mostarda C.T. (2020). Acute effects of resistance exercise with blood flow restriction in elderly women: A pilot study. J. Aging Phys. Act..

[41] Libardi C.A., Chacon-Mikahil M.P., Cavaglieri C.R., Tricoli V., Roschel H., Vechin F.C., Conceição M.S., Ugrinowitsch C. (2015). Effect of concurrent training with blood flow restriction in the elderly. Int. J. Sports Med..

[42] Conceição M.S., Gáspari A.F., Ramkrapes A.P.B., Junior E.M.M., Bertuzzi R., Cavaglieri C.R., Chacon-Mikahil M.P.T. (2018). Anaerobic metabolism induces greater total energy expenditure during exercise with blood flow restriction. PLoS ONE.

[43] Takarada Y., Takazawa H., Sato Y., Takebayashi S., Tanaka Y., Ishii N. (2000). Effects of resistance exercise combined with moderate vascular occlusion on muscular function in humans. J. Appl. Physiol..

[44] Park S., Kim J.K., Choi H.M., Kim H.G., Beekley M.D., Nho H. (2010). Increase in maximal oxygen uptake following 2-week walk training with blood flow occlusion in athletes. Eur. J. Appl. Physiol..

[45] Loenneke J.P., Wilson J.M., Marín P.J., Zourdos M.C., Bemben M.G. (2012). Low intensity blood flow restriction training: A meta-analysis. Eur. J. Appl. Physiol..

[46] Rossow L.M., Fahs C.A., Sherk V.D., Seo D.I., Bemben D.A., Bemben M.G. (2011). The effect of acute blood-flow-restricted resistance exercise on postexercise blood pressure. Clin. Physiol. Funct. Imaging.

